# Setting up research infrastructure for secondary use of routinely collected health care data in Croatia

**DOI:** 10.3325/cmj.2017.58.327

**Published:** 2017-10

**Authors:** Kristina Fišter, Borna Pleše, Ivan Pristaš, Tobias Kurth, Mirjana Kujundžić Tiljak

**Affiliations:** 1 University of Zagreb School of Medicine, Andrija Štampar School of Public Health, Department of Medical Statistics, Epidemiology and Medical Informatics, Zagreb, Croatia; kfister@snz.hr; 2 Croatian Institute of Public Health, Division of Health Informatics and Biostatistics, Zagreb, Croatia; 3 Institute of Public Health, Charité – Universitätsmedizin Berlin, Berlin, Germany

Secondary use of routinely collected health care data has for long been promising to provide better and less costly care (1). Defined as the use of clinical routine data outside direct patient care, secondary use can have many and varied objectives, for example, improvement of patient safety, identification of “best practice” approaches, conducting clinical research, continuous quality measurements and reports, identification of room for quality improvement, optimization of clinical documentation and its supporting systems, public health tasks, business management, accreditation, identification of market potential and commercial use, fraud detection and prevention, surveillance of adherence to guidelines and standards, compliance control, teaching and training, and disease, case, and therapy management (2).

To what degree the promise of secondary use has been fulfilled varies widely across settings. In Croatia, the newest member of the European Union with a population of about 4.3 million and universal health care coverage, a large number of registries have been set up over the past several decades (3). Recently, a uniform software platform has been developed and implemented for easier and more reliable data management across these registries. In addition, since 2009, all data generated in primary care and later on also in pharmacies have been deposited in the Central health care information system and warehouse, with reportedly over 99% coverage of general practitioner (GP) practices (4). Information from secondary care followed suit in the autumn of 2016 (5). All prescriptions and referrals are now also electronic. The Central electronic health record (eKarton), which was introduced in 2016 for all residents as part of the Central health care information system, thus now captures information from the whole spectrum of the health care system. Access is granted to patients to whom the records pertain, and each patient can reportedly grant access to physicians involved in their care (5).

All health care providers, public and private, create and send data to the national registries and databases, or create reports on their activities. The primary data sources in this context are all health care providers in Croatia including GP practices, hospitals, health care centers, and private care providers. The recipients of the data are the Health insurance information system (for financial reimbursement and health insurance purposes), the Central health care information system (as the main data exchange system at a national level), and the National public health information system (a platform that hosts all public health registries, including surveillance and reporting services). As unique identifiers have been compulsory in Croatia for forty years for all citizens, all these information sources can also be indirectly or directly linked to national registries, databases, reports, and international sources where relevant. It is, therefore, possible to use these linked data to provide policy makers and other stakeholders with new and useful insights based on analyses of big health care data (6). It has also been argued that the country’s size may be suitable for whole population analyses (Paul Taylor, Farr Institute London, personal communication, April 20, 2016).

It is apparent that routinely collected data in the Croatian health care system have become abundant and well supported by the information and communication technology. These health information systems have been successful in fulfilling their primary purpose, however, attempts at secondary use have been patchy. To our knowledge, secondary use as related to the Croatian setting has previously been addressed rarely and only tangentially (7-9). In other words, Croatia has for now been missing out on the opportunities that analyses of large data sets hold for health care services research and other research and policy informing purposes. Consequently, we have been missing out on the potential to improve areas such as assessment of patient outcomes and comorbidities, rationalization of the health care system, disease surveillance, evaluation of health interventions, evidence generation for health technology assessment, clinical quality monitoring, and syndromic surveillance. Such information could be gathered through robust analyses of large data sets acquired by linking various types of data sources including different types of patient registries mutually (eg, disease, services and product based) or with other data sources (eg, electronic health record, insurance data, prescriptions, laboratories), administrative data (eg, demographic and spatial data), process data (eg, time to care), and health care resources data (eg, personnel, use of high cost equipment, capacities and occupancy, waiting lists).

In order to fulfil the potential of secondary use, forming of a panel of independent experts and comprising all relevant stakeholders at the county and national level would be beneficial. The importance and depth of issues at stake make us propose that the Ministry of Health should take on the lead role in providing a forum for all relevant stakeholders and experts to discuss and jointly develop a secondary use infrastructure. Similar previous calls made in an attempt to improve eHealth practices in Croatia have for now been left unanswered (10).

One issue to solve is variation in data quality and data access procedures, which are not unified throughout the system at a national level. We believe both are key prerequisites for successful secondary use. Suboptimal coding practices require much regulation and education at all levels of the health care system, but they have not as yet received appropriate attention from the governing bodies. Another issue that hampers secondary data use is the low level of visibility of available data, especially for use by interested researchers.

A further matter to decide on is development of a strategy for validation of the data sources we have described for use in research. Various approaches to this end have been proposed (11). As data quality is essential in secondary use and may prove the main limitation, multiple carefully done validation studies are needed to ensure data quality in all dimensions, ie, accuracy, completeness, interpretability, relevance, timeliness, and coherence (12). Whether to adopt the Canadian model (13) or perhaps Swedish (14,15) or another is a matter for all stakeholders to decide jointly, since concerted efforts are sure to result in better use of the sparse resources as well as a better quality product.

The current legislation in Croatia lacks a clearly defined framework that would enable secondary use in line with the best practices, policies, and standards in eHealth (16). For example, the concept of secondary data use has not yet been incorporated in Croatian legislation. Furthermore, much remains to be done to educate researchers, policy makers, and the public about the potential benefits as well as pitfalls of secondary health care data use. Especially sensitive may prove views of the public on what should or should not be done in the name of secondary use. Experiences of others could help us avoid repeating mistakes seen in other settings (17,18). It should also be noted that our efforts to build a research infrastructure for secondary use come at a time when the data protection reform in the European Union has resulted in clearer regulation which is to come into effect in May 2018 (19). This is likely to help remedy some of the problems seen elsewhere previously but will also put additional requirements on our design of the secondary use infrastructure.

Given the setup of nationwide collected and available health care data, which exceeds the setting of many other European countries, it has to be argued at this point that it is unethical not to tap into the wealth of these data to the benefit of improving policy and clinical decisions, as well as improving research and academic infrastructure in Croatia. We believe time is ripe for ensuring meaningful use of these abundant and varied data. Concocted multi institutional efforts are needed in Croatia to set up quality infrastructure for a reliable, valid, and ethical secondary use of routinely collected health care data.

## References

[1] Safran C, Bloomrosen M, Hammond WE, Labkoff S, Markel-Fox S, Tang PC (2007). Toward a national framework for the secondary use of health data: an American Medical Informatics Association White Paper.. J Am Med Inform Assoc.

[2] Hackl WO, Ammenwerth E (2016). SPIRIT: Systematic planning of intelligent reuse of integrated clinical routine data. a conceptual best-practice framework and procedure model.. Methods Inf Med.

[3] Croatian Institute of Public Health. Registries and databases [in Croatian]. Available from: http://www.hzjz.hr/sto-radimo/statistika/registri-i-baze-podataka/. Accessed: November 5, 2016.

[4] Croatian Health Insurance Fund. CEZIH [in Croatian]. Available from: http://www.cezih.hr/index.html. Accessed: November 5, 2016.

[5] CEZIH. E-Chart [in Croatian]. Available from: http://www.cezih.hr/eKarton.html. Accessed: November 5, 2016.

[6] Schneeweiss S (2014). Learning from big health care data.. N Engl J Med.

[7] Kralj D, Kralj D. The Problems of designing a formal methodology of measuring status and quality of eHealth in Croatian primary care [in Croatian]. Zbornik radova IV. kongresa Koordinacije hrvatske obiteljske medicine. Šibenik, Croatia; 2013.

[8] Pajić V, Pristaš I, Meglič M (2013). Forming of a registry of European registries in health care - service oriented approach. Acta Med Croatica.

[9] Čizmić J (2009). Right of access to data in the medical documentation. Zb Prav Fak Sveuc Rij.

[10] Croatian Academy of Medical Sciences. Declaration on eHealth [in Croatian]. Available from: http://www.hcjz.hr/index.php/hcjz/article/view/25. Accessed: November 5, 2016.

[11] Weiskopf NG, Weng C (2013). Methods and dimensions of electronic health record data quality assessment: enabling reuse for clinical research.. J Am Med Inform Assoc.

[12] Cross-Border Patient Registries Initiative. PARENT Guidelines. Available from: www.parent-ror.eu. Accessed: November 5, 2016.

[13] Canadian Institute for Health Information. The CIHI Data Quality Framework, 2009. Available from: http://www.cihi.ca/CIHI-ext-portal/pdf/internet/data_quality_framework_2009_en. Accessed: November 5, 2016.

[14] Ludvigsson JF, Almqvist C, Edstedt Bonamy A-K, Ljung R, Michaëlsson K, Neovius M (2016). Registers of the Swedish total population and their use in medical research.. Eur J Epidemiol.

[15] Ludvigsson JF, Otterblad-Olausson P, Pettersson BU, Ekbom A (2009). The Swedish personal identity number: possibilities and pitfalls in healthcare and medical research.. Eur J Epidemiol.

[16] World Health Organization. Legal frameworks for eHealth. Global observatory for eHealth series - Volume 5. Geneva: WHO; 2012.

[17] McCartney M (2014). Do patients know that their records aren’t private?. BMJ.

[18] Godlee F (2016). What can we salvage from care data?. BMJ.

[19] European Commission. Reform of EU Data Protection Rules. Available from: http://ec.europa.eu/justice/data-protection/reform/index_en.htm. Accessed: September 24, 2017.

